# A “Tumour Safari” in East and Central Africa

**DOI:** 10.1038/bjc.1962.43

**Published:** 1962-09

**Authors:** Denis Burkitt


BRITISH JOURNAL OF CANCER

VOL. XVI SEPTEMBER, 1962 NO. 3

### A "TUMOUR SAFARI" IN EAST AND CENTRAL AFRICA

DENIS BURKITT

*From the Department of Surgery, Makerere College Medical School, and Mulago Hospital, Kampala, Uganda*

Received for publication May 4, 1962

FIVE years ago it became evident that the majority of malignant neoplasms observed in children in Uganda were but differing manifestations of a single distinctive tumour syndrome. The most frequent and characteristic presenting feature of this condition is a tumour involving one or more quadrants of the jaws. Other sites where tumours are commonly found include the kidneys, adrenals, ovaries, liver, thyroid, heart, intestine and the extra-dural space in the spinal canal.

The clinical and some of the epidemiological features of this tumour syndrome have already been described (Burkitt, 1958; Burkitt and O'Connor, 1961; Burkitt and Davis, 1961; Burkitt, 1962*a*, 1962*b*). O'Connor and Davies (1960) identified this tumour as a malignant lymphoma, and O'Connor (1961) has discussed the pathological and histological features.

Shortly after the syndrome was recognized as a specific clinical entity, it became evident that its distribution in Africa was limited.

Since the strikingly distinctive jaw tumours cannot readily be mistaken for any other lesion, these were used as an index to try to determine the limits of tumour distribution. An illustrated leaflet depicting the characteristic features of these jaw lesions, and containing a brief description of the other presenting features of this syndrome, was circulated to medical units throughout Africa. It was accompanied by a questionnaire asking the recipients whether they had observed these tumours in the areas where they had worked.

The information received indicated that the tumour was recognised right across tropical Africa from the coast of Kenya and Tanganyika in the east to the coast of Senegal in the extreme west. It was unknown in South Africa and north of the Sahara (Fig. 1). The Rhodesias appeared to be virtually exempt and very significantly the tumour was quite unknown in the heavily populated off-shore islands of Zanzibar and Pemba, although common on the adjacent Tanganyika coast (Burkitt, 1962*a* and *b*).

It became evident that the area of tumour distribution constituted a belt across tropical Africa with a tail running down the East coast. Moreover there were areas within this belt where, in spite of dense populations, the tumour did not exist. More detailed examination of tumour distribution in Uganda and Kenya indicated that the limiting factor in distribution pattern was an altitude barrier at about 5000 ft. above sea level. Haddow (1961, personal comunication), the Director of the East African Virus Research Institute at Entebbe, pointed out that the map of tumour distribution in Africa as we then knew it, corresponded to certain climatic factors.

It was considered that an effort to define as accurately as possible some portion of the apparent boundary of the " tumour belt " might provide valuable clues as to the aetiology of this condition. This exercise would be comparable to the pathologists choice of marginal tissue for detailed histological examination of any lesion.

FIG. 1.—Known distribution of the tumour in Africa including information obtained on this safari.

In choosing an area for closer examination, the whole northern boundary was considered impossible as the tumour would naturally disappear where populations dwindled into areas of virtual desert. The sea coast was the southern boundary in West Africa, and further east the border traversed Angola, a country obviously unsuitable for detailed medical investigation.

It was therefore decided to explore the southern limits of the eastern end of the belt, including the coastal tail.

The objective was to visit as many hospitals as possible along the " negative " side of the assumed " edge " and then along the " positive " side. These units were circulated nearly a year in advance so that the staffs might look out for these lesions and refer to their Hospital records.

This form of investigation, touring the African bush in a Ford station wagon that had already seen eight years of service in the Congo, is foreign to accepted concepts of cancer research, but has nevertheless proved fruitful.

With two friends, Dr. E. H. Williams and Dr. Clifton Nelson, 56 hospitals were visited and the staffs of many more reached through lectures and personal contacts. Moreover, since most of the doctors were in Government service, they had worked in different areas and so provided information on places we were unable to visit. We travelled in ten weeks over ten thousand miles through nine countries (Fig. 2). The journey was mostly by road but occasionally on goods trains and once by lake steamer, when roads were rendered impassable by a period of unprecedented rain.

FIG. 2.—The safari route.

At every hospital visited, we endeavoured to portray, with the aid of an album of illustrations, the various features of this tumour syndrome affecting children. Our purpose was to discover the localities from which these patients came, rather than to determine, in which hospitals the condition had been recognized.

### FINDINGS

These will be summarized rather than referred to in detail. The places from which patients had come are marked in Fig. 3.

### *Tanganyika*

With the exception of the Southern Highlands and areas depopulated from tsetse infestation, the tumour has been recognized over most of this territory.

The highest concentration appears to be round the south east shores of Lake Victoria. A seventy bed Mission Hospital south of Mwanza, recorded six cases in three months.

*Northern Rhodesia*

Evidence of the tumour was found only in the Luapula, Luangwa and Zambezi valleys, and in the relatively low country of Barotsiland round the upper

FIG. 3.—Map of East and Central Africa. All areas above 3000 ft. are shaded. The crosses indicate areas from which tumour patients have been observed.

reaches of the Zambezi. No cases had been observed in the higher plateaus. Evidence of only two cases was found at Lusaka, and of another two in the copper belt, but all four had been domiciled in the river valleys referred to above.

*Nyasaland*

The tumour is common along the lake shore and in the low country surrounding the Shire river south of the lake. Five patients had been admitted to a 60 bed hospital in six months. There was no evidence of its occurrence in the highlands along the western half of the territory.

*Southern Rhodesia*

Extensive enquiries revealed evidence of only eight patients suffering from this tumour. Two had been seen in Umtali, both of whom had come from the Sabi valley. Another two had been recorded at Salisbury, one from the Zambezi valley to the north, one from the Sabi valley to the south. Four had been recorded from Bulawayo, but one of these came from Northern Rhodesia. Of the others, one had come from the Zambezi valley and two from the northern part of the Limpopo valley. The eighth had lived south of the western end of the Kariba Lake and had attended Livingstone Hospital in Northern Rhodesia.

FIG. 4.—Diagrammatic representation of known distribution of children's lymphoma syndrome in East, Central and South Africa, showing the decreasing altitude barrier as distance from the equator increases. Shading represents areas of potential occurrence of tumour.

*Mozambique*

The condition was very familiar to doctors at Beira and at Lorenco Marques. Dr. Praetes had detailed records of over 40 patients who had come from widely scattered parts of the country during the last five years.

*South Africa*

The condition is virtually unknown in South Africa. Only three cases have been recorded at Johannesburg and two of these were white children. It is believed that they visited the coast for annual holidays and investigations to determine the circumstances of these visits are being undertaken.

*Swaziland*

The condition is unknown in this country.

The tumour distribution in the Central African Federation does not correspond to population densities.

*Ruanda-Urundi*

This country, which lies almost immediately south of the equator, had been included in our original safari programme, but unprecedented rains rendered access by road impossible. The population density of over 200 per square mile is one of the highest in Africa. The vast majority of the population live at an altitude above 5000 ft.

A recent visit revealed that only one doctor, working in Usumbura on the shore of Lake Tanganyika, at an altitude of 2500 ft. could remember having seen

Fig. 5.—Map of sub-equatorial Africa. Only on shaded areas do temperatures fall below 64° F. The crosses indicate areas from which tumour patients have been observed.

a case of this tumour. A nurse and a medical assistant stated that they had each seen one patient with a tumour similar to the illustrations shown them. No other evidence was forthcoming in spite of the fact that senior doctors have been watchful for this tumour for some years. It can therefore be concluded that the condition is virtually unknown among the two and a half million inhabitants of this small country.

*" Edges "*

In four areas fairly sharply defined " edges " to the tumour-bearing area were detected.

1. Although the tumour is particularly common round Lorenco Marques, it is totally unknown in Swaziland, less than 100 miles away, but at an altitude of 2000 ft.

2. All the medical staff at Beira were familiar with the condition, but only one case had been seen in 18 months at Villa Perry less than 100 miles west but at an altitude of 2000 ft. One hundred miles further west at Umtali (4000 ft.) the only two patients recorded had come from the Sabi valley to the south.

3. Although the tumour is relatively common around the northern shore of Lake Nyasa, at 1500 ft. it is unknown in the Highlands, north of Tukuyu, situated some 30 miles from the lake, at an altitude of over 4000 ft.

4. Tumour incidence is high at Kisumu and round the north east shore of Lake Victoria. It disappears as the land rises towards the Kenya Highlands.

### *Summary*

(Fig. 3, 4 and 5.)

1. In Uganda, Kenya and Tanganyika, the tumour can apparently occur anywhere except in areas over 5000 ft. above sea level.
2. In the Federation of the Rhodesias and Nyasaland, the condition is found only in the river valleys and on the lake shore.
3. The condition is widely recognized throughout the central plain of Mozambique as far south as the southern tip.
4. It is virtually unknown in South Africa.
5. It is virtually unknown in Ruanda-Urundi.

### CONCLUSIONS

1. The tumour is dependent on altitude (Fig. 3).
2. The critical altitude falls as the distance from the equator increases (Fig. 4).
3. Altitude is therefore considered to be only a limiting factor in so far as it reflects temperature.
4. The actual limiting factor appears to be a minimum temperature of about 60° F. (Fig. 5).

### SPECULATION

The fact that this unusual tumour is temperature dependent, implies that some vector may be involved in its transmission. This in turn suggests the possibility that a virus is implicated. This interpretation of the epidemiological features was first voiced to me by Professor J. N. P. Davies.

It is significant that the mosquito-born epidemics of O'nyong-nyong fever, which in recent years swept through Uganda, Southern Tanganyika and Northern Nyasaland, were turned back at altitudes approximately corresponding to the upper limits of distribution of this tumour. This may point to comparable vectors transmitting the causitive agent in these two conditions.

### SUMMARY

The geographical distribution of a children's lymphoma syndrome common in tropical Africa has been examined.

The altitude determining tumour occurrence varies with latitude, and a minimum temperature appears to be the actual factor limiting the tumour distribution.

I am particularly anxious to thank the Medical Officers. both Government and Mission, in the territories we visited for their most helpful co-operation, and at the same time to pay tribute to the many who do so much over and above the requirements of duty to advance medical knowledge. The list is so great that I have purposely omitted names.

I am most grateful to the Chief Medical Officer, Uganda, and the Professor of Surgery at Makerere University College Medical School, for permission of absence from duty to do this investigation.

I wish to express sincere thanks to the Medical Research Council and the Department of Technical Co-operation, London, who provided the necessary funds, and to the Sloan Kettering Institute and the British Empire Cancer Campaign who had made previous donations to this study.

### REFERENCES

Burkitt, D.—(1958) *Brit. J. Surg.*, **46**, 218.—(1962*a*) *Post Grad. med. J.*, **38**, 71.—(1962*b*) *Ann. R. Coll. Surg.Engl.*, **30**, 211.

*Idem* and Davies, J. N. P.—(1961) *Med. Pr.*, **245**, 367.

*Idem* and O'Connor, G. T.—(1961) *Cancer*, **14**, 258.

O'Connor, G. T.—(1961) *Ibid.*, **14**, 270.

*Idem* and Davies, J. N. P.—(1960) *Pediatrics*, **56**, 526.


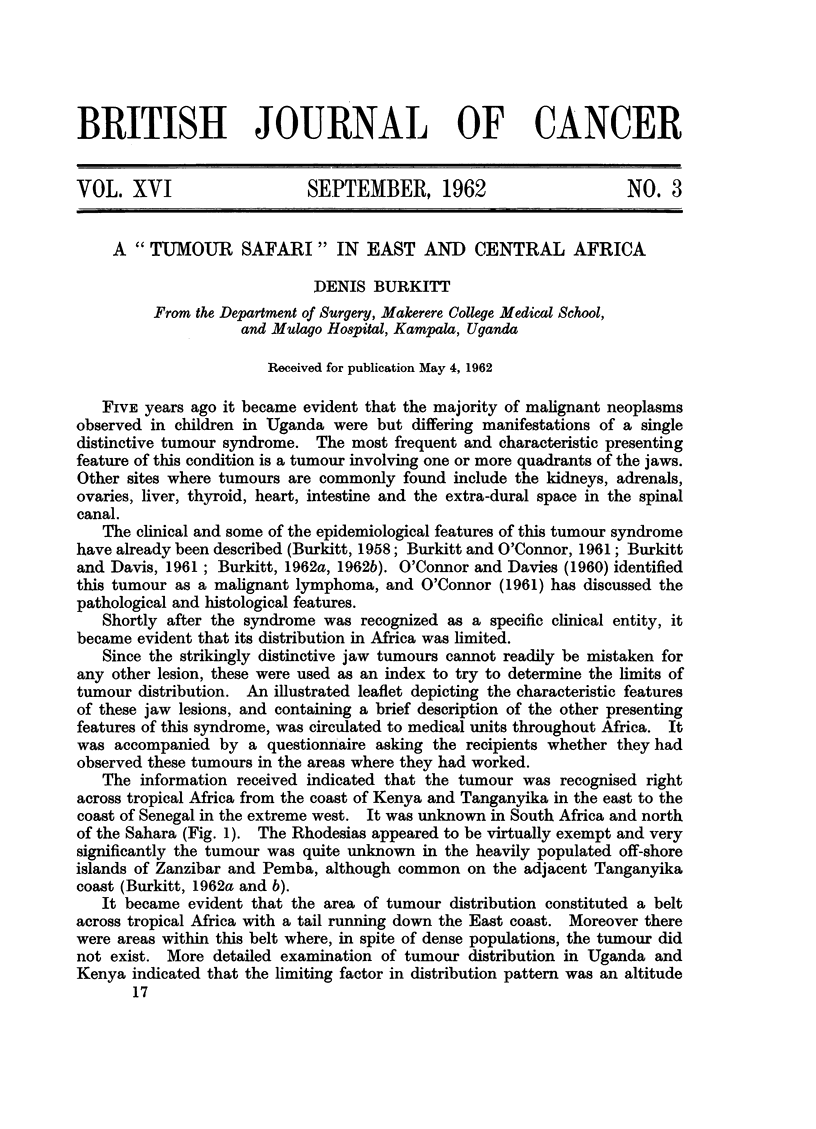

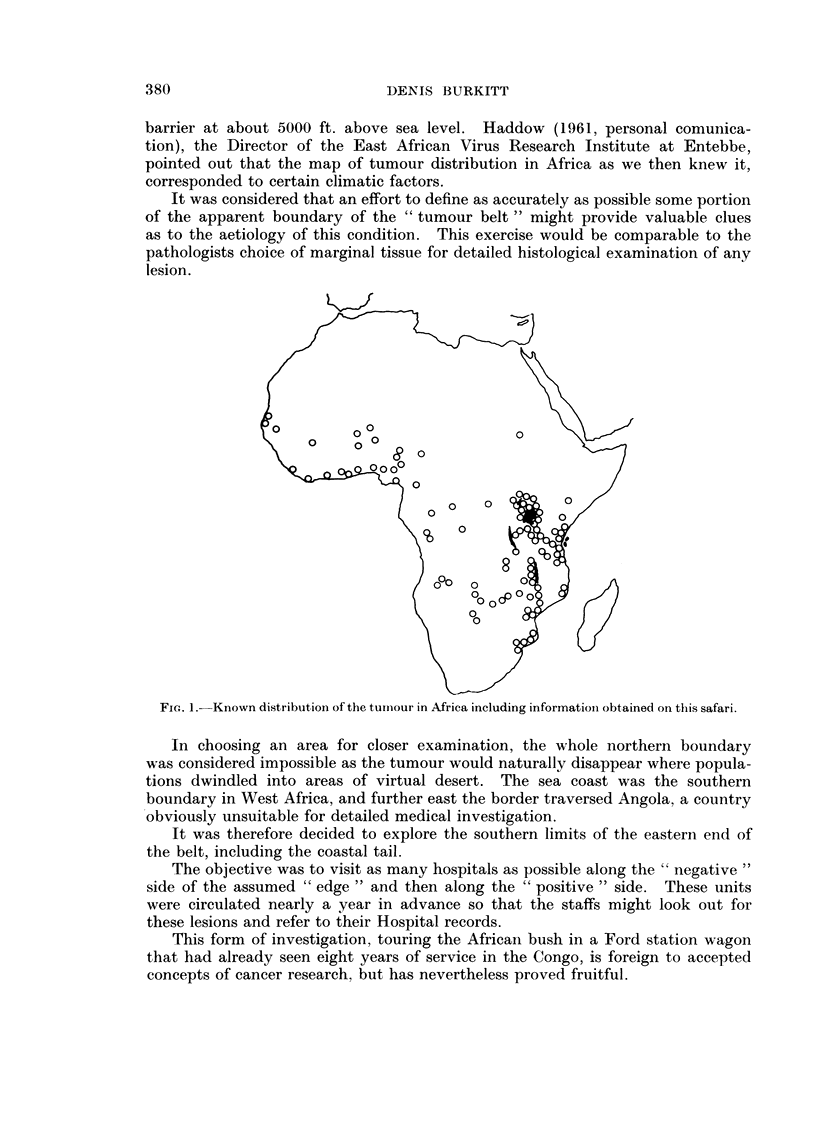

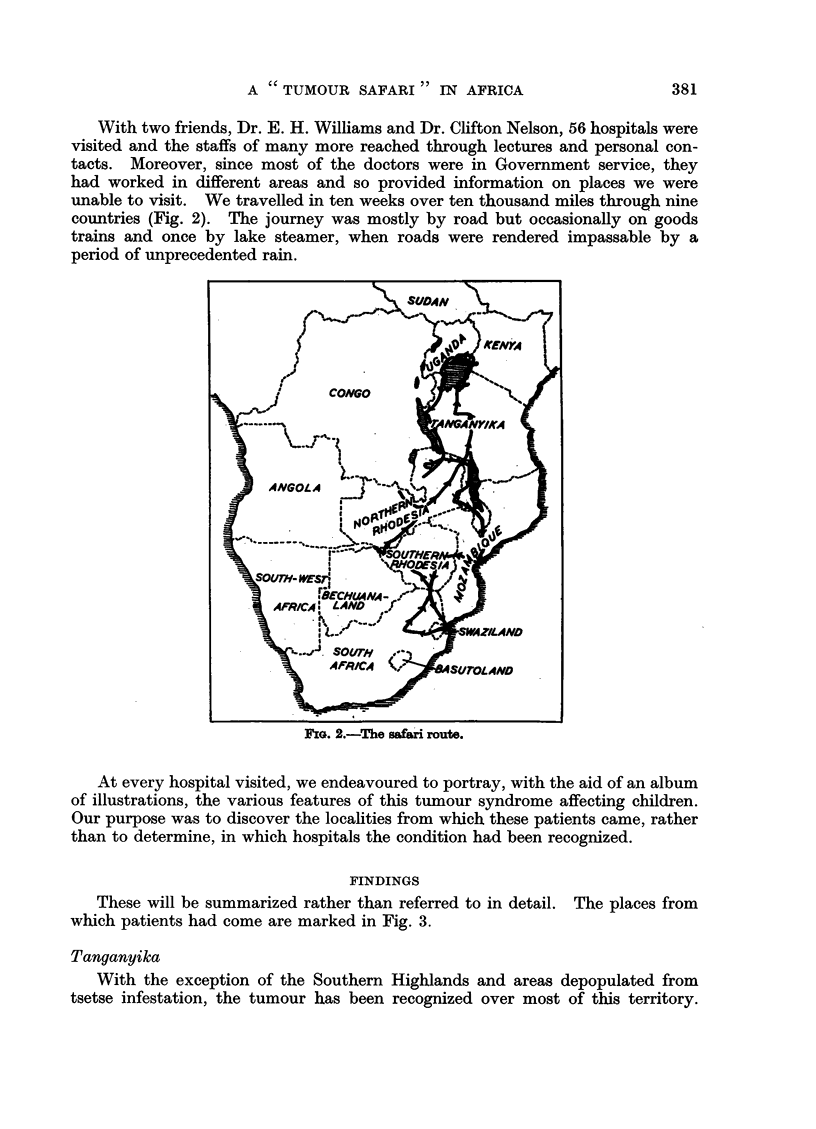

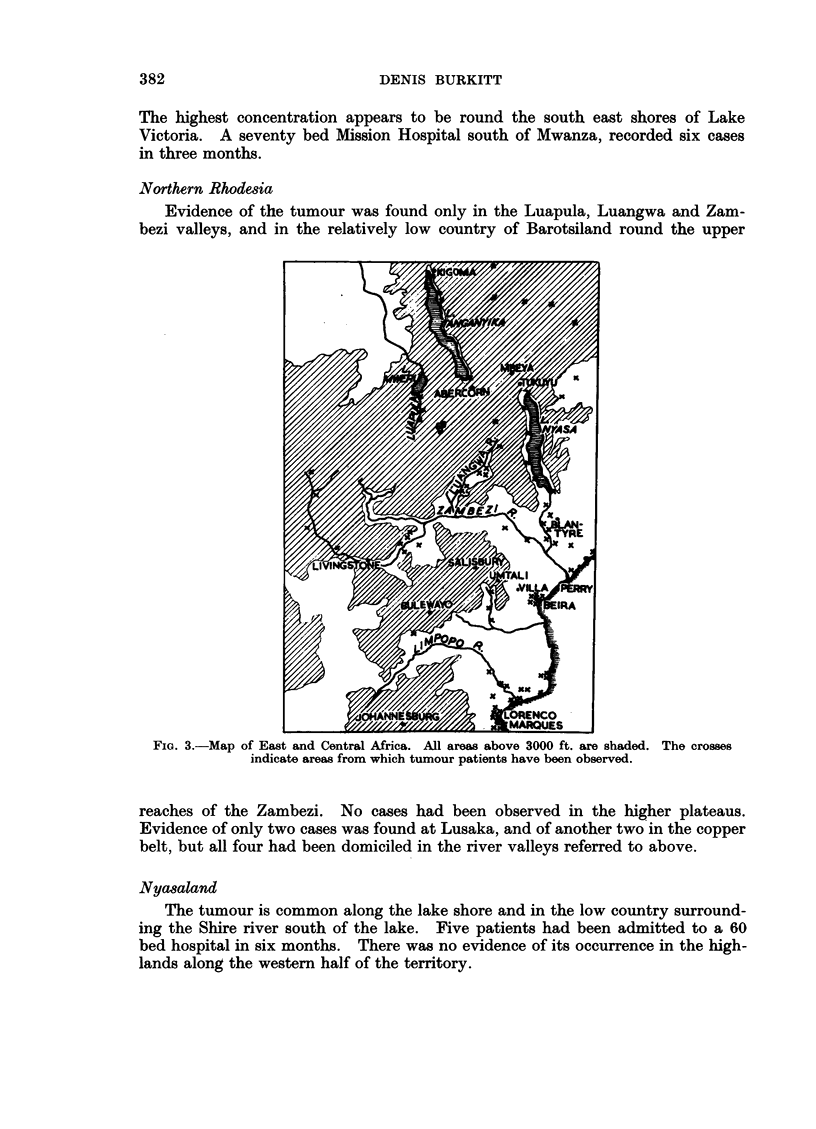

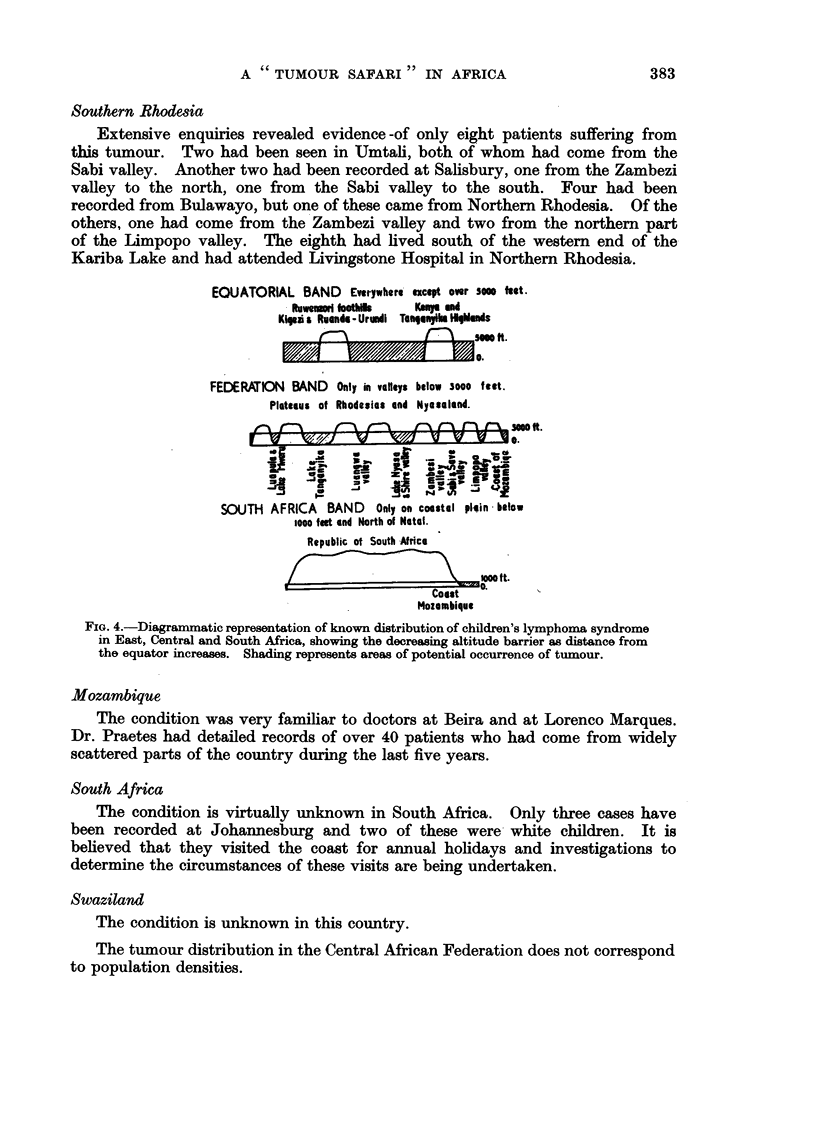

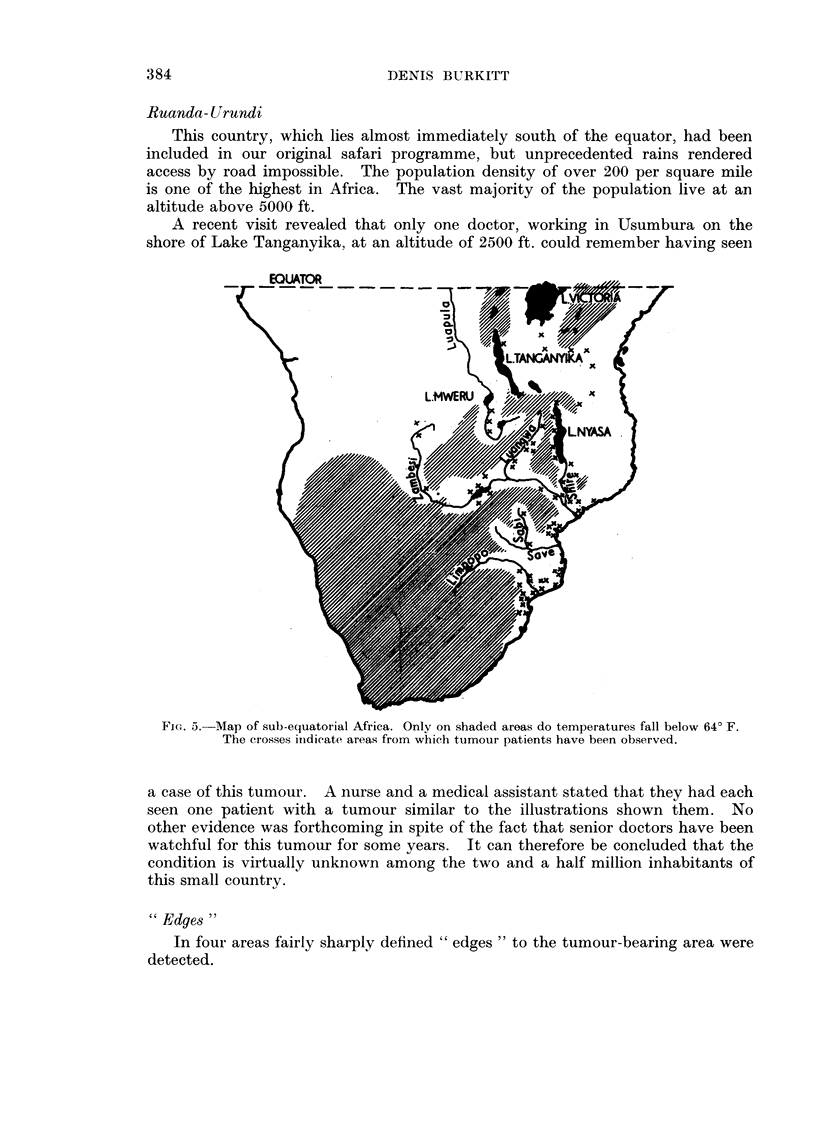

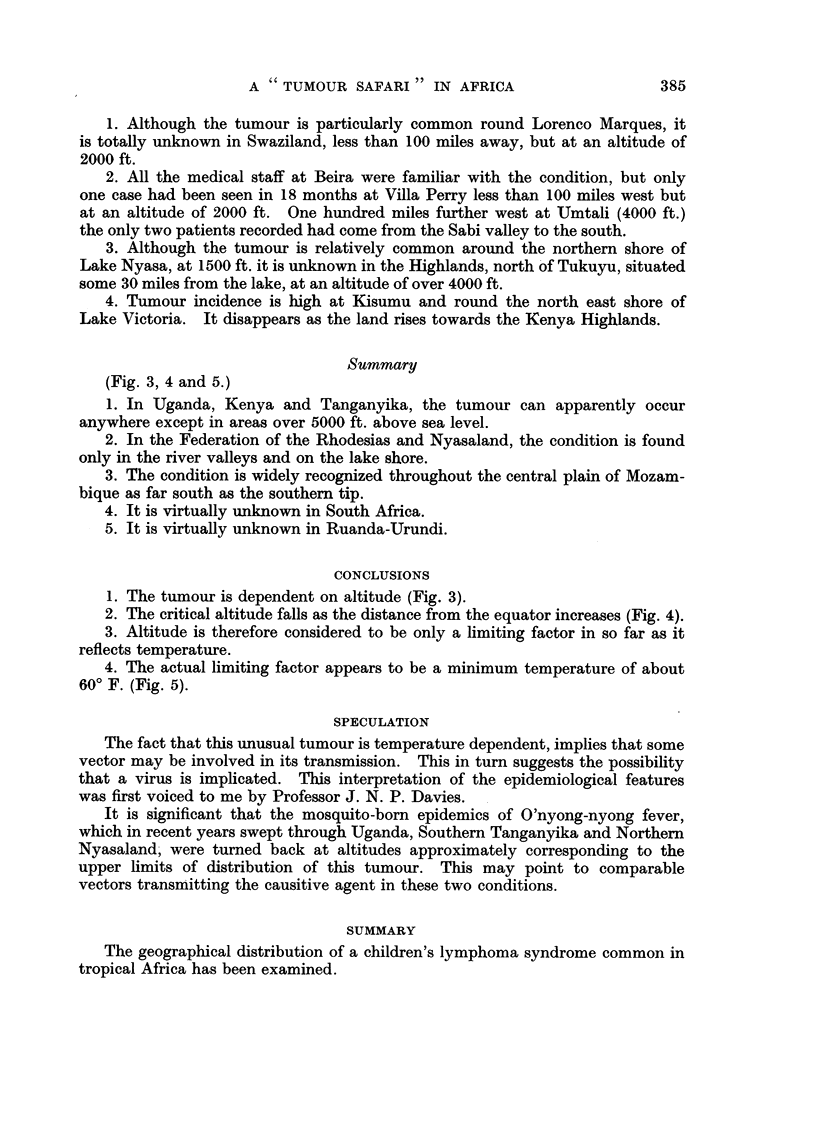

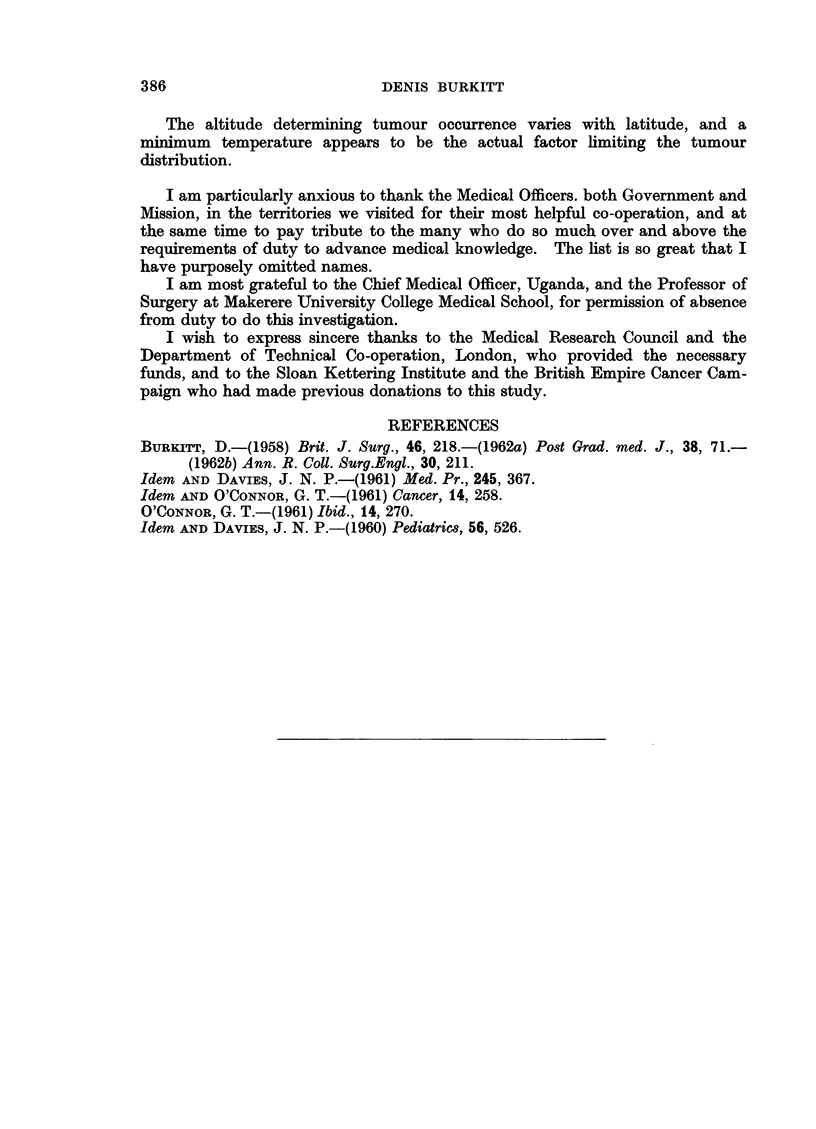


## References

[OCR_00340] BURKITT D. (1962). A lymphoma syndrome in African children.. Ann R Coll Surg Engl.

